# Farmers' suicide: Across culture

**DOI:** 10.4103/0019-5545.58286

**Published:** 2009

**Authors:** P. B. Behere, M. C. Bhise

**Affiliations:** Department of Psychiatry, Mahatma Gandhi Institute of Medical Sciences, Sevagram - 442 102, District Wardha, Maharashtra, India

***“Indian Agrarian Crisis: Farmer, the most endangered species”***

Despite the popular image of farming as a peaceful and healthy way of life, agriculture has the highest rates of mortality in any industry.[1] Suicide among farmers is now a universal phenomenon. Studies across the globe have identified farming as one of the most dangerous industries. Farming environments are characterized by a broad and changeable range of physical, biological and chemical hazards that are similar across all cultures. Thus, it is important to view the issue of farmers' suicide from a global perspective.

## CONTEXT OF FARMING

Although farming practices, production systems and type of farms are diverse, there are commonalities across the farms, which are important to health. Most farms continue to be family-owned and operated business and are exposed to the volatility of commodity markets, the variability of weather patterns and the influence of respective government regulations.

Farmers are thus exposed to a high rate of stress. Physical stressors and hazards of the farm environment are compounded by regulatory framework and economic dynamics of managing farm business. These operate in the context of declining trends of trade for agricultural produce, volatile commodity markets, limited availability of off-farm employment, growing cost of machinery and production and loss of farm or livelihood due to crop failures.[2] Economic concerns and government bureaucracy have been consistently identified as a major cause of stress and a contributor to suicide. There is no customary or mandatory retirement age for farmers all over the world and many tend to work beyond the customary retirement age, placing the younger generation in a dependant relationship with their parents for much longer than is typical. This can lead to tension between the two generations on the farm. Recently, we have witnessed such cases in India: The son becoming alcoholic following the father having retired from active government service and taking a dominating and leading role in farming. Roles between work, home and family are often blurred, with farming operating as an occupation and way of life for many farmers. Research had shown a relationship between monetary and family problems with suicide. British farmers were more concerned about family problems[3] while indebtedness and monetary concerns were reported to be the major reason for suicide among Indian farmers.[4,5]

## FARMERS' SUICIDES IN DIFFERENT CULTURES

Studies in India, Sri Lanka, USA, Canada, England and Australia have identified farming as one of the most dangerous industries associated with a high suicide rate than in general population. In India, farmers' suicides had been reported from various states, viz. Punjab, Maharashtra, Andhra Pradesh, Kerala and various other states with varied cultural practices and farming patterns. A study in the Vidarbha region of Maharashtra had associated indebtedness (87%) and deterioration in the economic status (74%) as major risk factors for suicide.[6] This study has revealed that age-adjusted suicide mortality rate for male farmers had trebled from 17 in 1995 to 53 in 2004. An independent study by the author in the region concluded that farmers committing suicide were in their 40s, who were living with family and most were married. Among modes of suicide, poisoning was the most prevalent, followed by hanging and jumping from a height.[7] In England and Wales, in contrast, fire arms were the method most frequently used by male farmers, followed by hanging and carbon monoxide poisoning. Farmers who commit suicide tend to use methods to which they have easy access because of their occupation. In India, due to the easy availability of pesticide and lack of education and efforts on the part of the system to train farmers in safe use of it, pesticide consumption is the most common method of committing suicide.[7,8] On the other hand, fire arms are not easily available and affordable due to the high cost. Reports from the state of Punjab showed that a majority of the victims were small and marginal farmers who were loners and heavy drinkers or drug users and 20% had informed relatives or friends about their suicidal intent. In Australia and the United Kingdom, male farmers have higher suicide rates than the national average and a higher rate of suicide than other rural males.[9] Farmers in the UK were shown to have a lower incidence of mental illness and used fire arms as a method of suicide. A decline in farmer's suicide was recorded after introduction of legislation on fire arm purchase, storage and registration in 1989 in England, indicating the role of easy accessibility of dangerous means and rate of suicide. Even in the absence of psychiatric morbidity, farmers were more likely to report that life is not worth living compared with the general population[10] and suicide in them was an end point to a series of difficulties that accumulated over time. In Australia, there is a strong correlation between draughts and suicide rates among farmers. In the US, there was a rise in farmers' suicide after great depression. To counter this, the government started a farmers' insurance program, which is the only major federally managed insurance program, except Medicare in the United States. When compared with Australia, India is grossly, socially and geographically different, but shares farmers' suicide alike. Scarcity of water in Australia is due to successive draughts while in India it is mainly due to political reasons. Migration to urban areas and shrinking of farm employment are the other stated causes while in India lack of credit and cost of raising genetically-modified crops are the reasons sought. Political response to this issue is also grossly different in the two nations. In Australia, in foresight of draught, there is rapid mobilization of social workers, psychologists and psychiatrists to the draught-hit region along with other supportive measures while in India it is predominantly limited to political announcement of ex-gratia benefits and not toward prevention strategies.

## FEMALE FARMERS SUICIDES

Suicide among female farmers is on the rise. Studies of women in farming have found high levels of stress, fatigue and depression.[11] Explanations most commonly given are role conflicts and high work load. Farm women, unlike men, experience stress not only due to the farm operations but also due to the impact of farming stressors on the physical, social and financial wellbeing of all family members. As farming has become less profitable, women are taking more and more on- and off-farm work to supplement the family income. Farming women in this position often become stressed and fatigued due to multiple tasks and the conflict between their traditional role as a housemaker and the need for off-farm income. The female farmers have an additional burden of performing household chores apart from farming. This makes farm women a high risk category for suicide. England, Australia and now India had reported suicide among this gender.[12]

## WAY AHEAD

Farming is clearly a high-stressed and dangerous occupation. Changing farming practices and various regulations governing it have compounded stressors traditionally associated with agricultural production. A number of studies have found high rates of depression and anxiety among farmers. Various risk factors act in cohesion to culminate into suicide by farmers. Although there are social and geographic variations, farmers' suicide is a global problem that needs detailed evaluation. The preventive strategies used by other nations may not completely apply in the Indian scenario, but it can be a guiding path for more research and formulation of preventive strategies. Farmers face a particular set of issues related to access to health care. Severe maldistribution of psychiatrists and psychologists among the rural and urban population is a global problem and needs to be addressed by effective government strategies, which may include incentive-based programs as run by Australia. Active participation by psychiatrists in prevention and research work in the field of farmers' suicide is now being recognized in India.[12,13] Heightened visibility among small farming communities may lead to fear of stigma related to seeking psychiatric services. This area needs to be investigated further. In India, 40% of the farmers would want to quit agriculture and take up some other career as a part of insecurity.[7] Work done in south Indian states like Andhra Pradesh and Karnataka is a success story in field of mental health, but much more needs to be done urgently by us professionals for containment of this issue. It is high time now to take necessary steps otherwise we may be facing extinction of another group from earth, this time a class from our own *homo*-*sapiens* species, our food growers - *the farmers*.
